# Pressureless Crystallization of Glass for Transparent Nanoceramics

**DOI:** 10.1002/advs.201901096

**Published:** 2019-06-28

**Authors:** Shaofei Wen, Yunpeng Wang, Bijiao Lan, Weida Zhang, Zhuo Shi, Shichao Lv, Yujun Zhao, Jianrong Qiu, Shifeng Zhou

**Affiliations:** ^1^ State Key Laboratory of Luminescent Materials and Devices School of Materials Science and Engineering South China University of Technology Guangdong Provincial Key Laboratory of Fiber Laser Materials and Applied Techniques Guangdong Engineering Technology Research and Development Censter of Special Optical Fiber Materials and Devices Guangzhou 510640 China; ^2^ Department of Physics South China University of Technology Guangzhou 510640 China; ^3^ College of Optical Science and Engineering Zhejiang University Hangzhou 310027 China

**Keywords:** crystallization, glass, nanoceramics, near‐/mid‐infrared luminescence, optical filtering

## Abstract

Transparent nanoceramics embedded with highly dense crystalline domains are promising for applications in missile guidance, infrared night vision, and laser and nuclear radiation detection. Unfortunately, current nanoceramics are strictly constrained by the stringent construction procedures such as super‐high pressure and containerless processing. Here, a pressureless crystallization engineering strategy in glass for elaboration of transparent nanoceramics and fibers is proposed and experimentally demonstrated. By intentional creation of a sharp contrast between nucleation and growth rates, the crystal growth rate during glass crystallization can be significantly suppressed. Importantly, this unique phase‐transition habit enables the achievement of transparent nanoceramics and even smooth fibers with extremely tiny crystalline size (≈20 nm) and high crystallinity (≈97%) under atmospheric pressure. This allows the generation of an attractive nonlinear optical response such as dynamic optical filtering and luminescence in the mid‐infrared waveband of 4300–4950 nm. These findings highlight that the strategy to switch the phase‐transition habit of glass into the unconventional crystallization regime may provide new opportunities for the creation of next‐generation nanoceramics and fibers.

## 1 Introduction

Transparent ceramics are a typical multiphase system in which crystalline grains are usually isotropic and intimately stacked with each other, allowing light to continuously propagate with negligible scattering. These material candidates are of great interest in a wide range of applications, including missile guidance, infrared night vision, and laser and nuclear radiation detection.^1^, ^2^, ^3^, ^4^, ^5^ Downsizing the grains to the nanoscale to create transparent nanoceramics is expected to offer the promise of material properties that could exceed the classic transparent ceramics. Despite substantial progress in ceramic technology, it has been challenging to prepare this type of unique transparent nanoceramics with nanosized grains. The current and primary approaches derived from powder sintering synthesis generally impose stringent control over a set of experimental variables, such as pressure, temperature, sintering duration, and these processes are always accompanied by undesired rapid grain growth.^6^, ^7^, ^8^ Alternatively, more extreme synthesis techniques, including super‐high pressure, spark plasma sintering, and containerless processing may shift the grains toward submicrometer region;^9^, ^10^, ^11^, ^12^, ^13^ however, the methods are inherently not scalable and also strictly limit the available sample size. Therefore, the development of new strategy for construction of transparent nanoceramics under mild conditions could bring about a new repertoire of ceramics.

Here, we propose a novel approach to construct transparent nanoceramics by engineering the phase transition of glassy phase. The strategy allows fine‐tuning of the size of crystal grains spanning three orders of length scales from ≈15 µm to ≈20 nm, via carefully manipulation of nucleation and growth habits of glass crystallization. Accordingly, transparent nanoceramics and the corresponding tiny fiber with smooth surface can be successfully fabricated at atmospheric condition. Various intriguing optical phenomena including smart radiation shielding and broadband near‐/mid‐infrared luminescence has been demonstrated in the transparent nanoceramics for the first time.

## 2 Results and Discussion

### 2.1 Material Design

**Figure 1:**
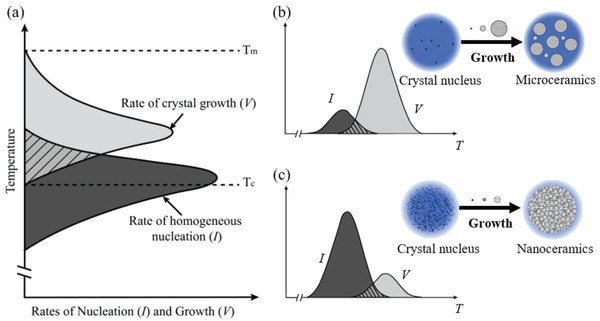
A schematic model illustrating the strategy for steering crystallization habit of glass. a) The classical curves of the temperature dependent nucleation and growth rate. b,c) Reshaping the crystallization rate curves via suppression or enhancement of nucleation rate leads to microcrystallization (b) or nanocrystallization (c), respectively.

The study focuses on the glass matrix because it intrinsically combines the unique features of supercooled liquid and solid state.^14^ On one hand, its crystallization can potentially enable the achievement of fully dense polycrystalline solids (i.e., totally absence of porosity), which has been recognized as a formidable challenge via conventional sintering approach. On the other hand, the derived supercooled solid exhibits strong temperature dependent viscosity and the crystallization starts to occur at extremely high viscosity state (typically ≈10^6^–10^10^ Pa s). As a result, each crystallization spot is position‐fixed and the corresponding structure evolution occurs independently, facilitating for creation of uniformly dispersed microstrucutre. To quantitatively describe the proposed strategy, the classical nucleation‐growth model and the microstructure evolution inside glass are correlated and schematically presented in **Figure** **1**. Figure 1a presents the characteristic curves for the nucleation (*I*) and growth (*V*) rates, in which the “low” and “high” temperature sections are mainly dominated by the kinetics and thermodynamics, respectively. Generally, the nucleation and growth curves are partially overlapped, and exhibit comparable and moderate magnitude in maximum rate. Thus, simple one‐step thermal activation usually leads to the uncontrollable crystallization and broad crystallite‐size distribution. Although multistep heat‐treatment process for separating the nucleation and growth stages may partially relieve this issue, realization of high‐volume nanocrystallization remains an arduous task. Keeping these limitations in mind, we devoted our efforts to reshaping the crystallization rate curves and supposed that tune of the rate contrast may switch the crystallization habit (Figure 1b,c). As an extreme case, we expected that significantly raising the nucleation curve and simultaneously suppressing the growth one may trigger dense nanocrystallization, leading to the formation of transparent nanoceramics (Figure 1c).

**Figure 2:**
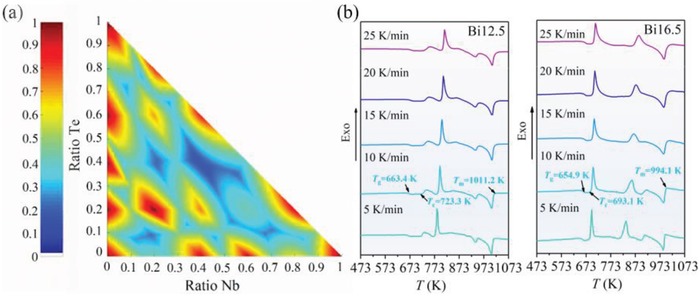
The crystallization habit of Te–Nb–Bi–O system. a) The probability distribution of single phase under different chemical potential. b) DSC curves of the glass samples under different reheating rates.

To validate our research idea, a typical tellurite multicomponent glass was selected as a prototype system because it has attracted much attention in a wide range of applications, such as broadband telecommunications, mid‐infrared spectroscopy, nonlinear optical processing, and acousto‐optic modulation.^15^, ^16^, ^17^ The material design principle mainly involves in the seeking appropriate hybrid elements that i) notably enhance the nucleation tendency and ii) constrain the crystallite's growth. For the first rule, we employed the field strength (*F*) as the key parameter because an increase in *F* value represents the enhancement in relaxation tendency. For the second criteria, we adopted diffusion energy barrier as the indicator because the creation of chemical environment with high diffusion energy barrier is believed to be the most effective way to constrain crystal growth. Based on these two fundamental criteria, we selected Nb and Bi as the hybrid elements because the former exhibits extraordinarily large field strength (*F*_Nb5+_ = 7.24) and the latter belongs to the heavy metal with large size (*r*_Bi3+_ = 0.117 nm) which may produce strong steric hindrance effect. As an added benefit, Nb/Bi and O can form unique structure units which are compatible with the –Te–O– networks. For example, the incorporation of Nb_2_O_5_ may provide O^2−^ that can trigger the transformation of [TeO_4_] trigonal bipyramid into [TeO_3_] or [TeO_3+1_] units, resulting in a more stable network structure.^18^ Based on the Te–Nb–Bi–O system, we first performed theoretical analysis to check the composition area where the single phase can be obtained. The calculation was carried out based on the grand canonical ensemble of thermodynamics and statistical physics and the results are highlighted in **Figure** **2**a. According to the calculation results, the area with the Bi/Nb atomic ratio between 0.8 and 3 was studied and two representative glasses with the composition of TeO_2_/Bi_2_O_3_/Nb_2_O_5_ = 75/12.5/12.5 (in mol%, denoted Bi12.5) and TeO_2_/Bi_2_O_3_/Nb_2_O_5_ = 75/16.5/8.5 (in mol%, denoted Bi16.5) were fabricated.

### 2.2 The Crystallization Behavior of Glass

The thermal properties of the as‐made glass matrix were systematically investigated for studying the thermodynamics and kinetics of crystallization process. Figure 2b presents the differential scanning calorimetry (DSC) curves with different reheating rates (*q*) of 5, 10, 15, 20, and 25 K min^−1^. *T*_g_ is the glass transition temperature measured at reheating rate of 10 K min^−1^; *T*_m_ is the melting temperature which is defined as the ending temperature of melting peak at reheating rate of 10 K min^−1^; *T*_c_ is the crystallization temperature which is calculated as the onset temperature of crystallization at reheating rate of 10 K min^−1^ (corresponding to the first exothermic peak in Figure 2b). Based on these characteristic values, the crystallization tendency of glass was examined and it can be correlated with the glass stability. The physical parameter used to evaluate the glass stability (*K*_H_) can be calculated according to the following equation^19^

(1) $$K_{H}=\frac{T_{c}-T_{g}}{T_{m}-T_{c}}$$

The *K*_H_ value of Bi12.5 and Bi16.5 was estimated to be 0.21 and 0.13, respectively. The notable decrease of *K*_H_ with the enhanced content of Bi indicates that Bi_2_O_3_ prefers to act as network modifiers to break the glass network structure. And the hybridization of Bi species reduces the stability of glass and promotes its crystallization tendency. It can be understood from the topological configuration of Bi‐related structure: Bi^3+^ ions are found in the form of [BiO_6_] and [BiO_3_] units in the glass matrix, which are believed to coordinate nonbridging oxygen and enhance the crystallization tendency (Figure S1, Supporting Information).^20^

**Figure 3:**
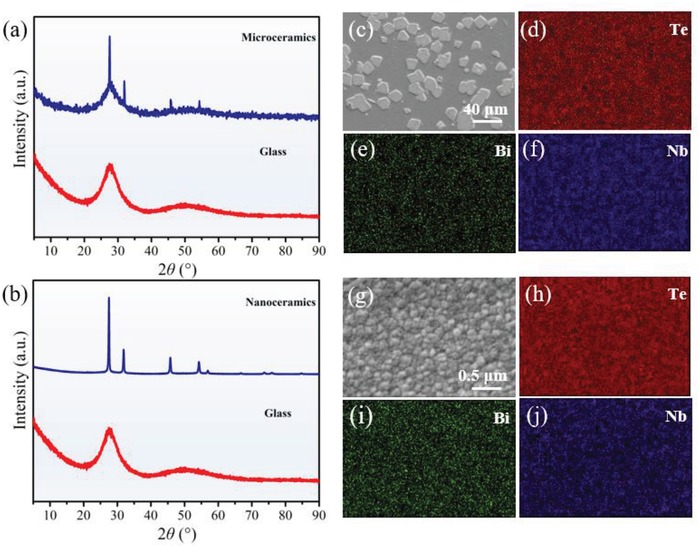
The microstructures of the microceramics and nanoceramics. a,b) XRD patterns of microceramics (a) and nanoceramics (b). c–j) SEM‐EDS mapping images of the microceramics (c–f) and nanoceramics (g–j).

To examine the detailed crystallization habit of glass phase, the structure analysis by X‐ray diffraction (XRD) and scanning electron microscopy (SEM) measurements was performed. The results show that with the increase of Bi concentration, the changes of microstructures are evident. The XRD characterizations (**Figure** **3**a,b) indicate the precipitated phase in sample Bi12.5 and Bi16.5 can both be well assigned to the Bi_0.8_Nb_0.8_Te_2.4_O_8_ phase which is isostructural to the β‐Bi_2_Te_4_O_11_ (PDF#00‐052‐0055).^16^ Notably, the crystallinity of sample Bi16.5 (≈97%) is about 7 times larger than that of Bi12.5 (≈14%). SEM images show that irregular particles with nonuniform and large size varying from 5 to 20 µm are sparsely distributed in sample Bi12.5 (Figure 3c). In stark contrast, the extremely dense and tiny particles are homogeneously precipitated in sample Bi16.5 and the size of particles can be tuned down to 20 nm (Figure 3g). Furthermore, energy‐dispersive spectrometer (EDS) examinations on the element distribution during crystallization process show the absence of concentration contrast between glass and crystalline phase, demonstrating the occurrence of homogenous crystallization (Figure 3h–j). The minimal difference of structure between glass and crystal and the shortest diffusive distance of particles during the congruent crystallization process is supposed to lead the slow crystal growth process (additional information about the structural evolution can be seen in Figure S2 in the Supporting Information).^21^ Taken together, above results unambiguously suggest the rational elemental hybridization approach is quite effective for regulating the crystallization habit of glass phase. Significantly, the strategy indeed enables triggering of the occurrence of the expected nucleation dominant crystallization habit proposed in Figure 1c and significantly, demonstrate the success in achievement of nanoceramics in sample Bi16.5.

### 2.3 Nanoceramics and Nanoceramics Fibers

**Figure 4:**
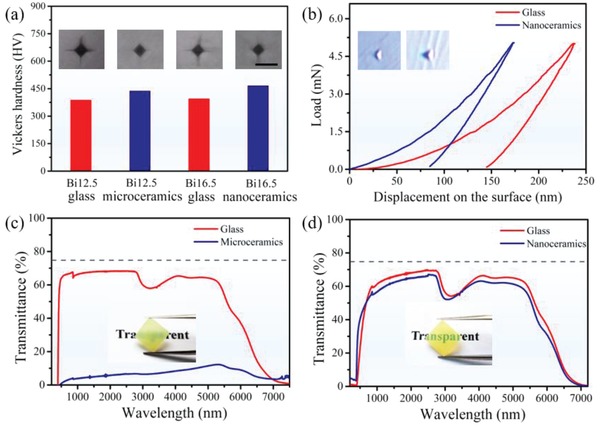
The mechanical and light transmission properties of the microceramics and nanoceramics. a) Vickers hardness and the corresponding images of indentation, the scale label in these images is 20 µm. b) The load–depth curves of the nanoceramics. The inset show the corresponding indentation. c,d) Optical transmission spectra of microceramics (c) and nanoceramics (d). The insets show the photographs of the samples. The gray dashed lines indicate the theoretical transmittance (≈75%) which is calculated with an average refractive index of 2.21 at λ = 632.8 nm.

The obtained nanoceramics are found to exhibit excellent mechanical and optical properties. The mechanical properties including hardness and elastic modulus of the samples were investigated by the Vickers hardness tester and nanoindentator equipped with a diamond Berkovich indenter. Significantly, the nanoceramics own the highest hardness (466 HV) which is higher than that of the microceramics (438 HV) and precursor glass (388 HV for Bi12.5 and 395 HV for Bi16.5). It can be directly observed in the micrograph of indentation (the inset of **Figure** **4**a), where the nanoceramics presents small size indentation and negligible crack propagation compared with the precursor glass and microceramics. The improvement should be associated with the considerably reduced crystal‐size and simultaneously enhanced crystallinity of nanoceramics.^22^ On one hand, nanoscale crystals imply larger volume fraction of the boundaries (compared with microscale crystals), resulting in the improved ability for hindering the propagation of the crack. On the other hand, the increase in crystalline volume fraction may lead to more compact microstructure, that is highly favorable for further improving the mechanical properties. The typical load curves as function of the displacement of nanoceramics are presented in Figure 4b. The results clearly indicate that a larger pressure is needed for nanoceramics to make the same indentation depth as the precursor glass. According to the load–depth curves, the hardness and elastic modulus are estimated to be 6.56 and 66.72 GPa for as‐made glass and 15.48 and 108.34 GPa for nanoceramics, respectively, further confirming a significant improvement in mechanical properties after high‐volume nanocrystallization.

The optical transmission features of the samples were investigated and the results are presented in Figure 4c,d. Surprisingly, nanoceramics present extraordinarily high transmittance over the broad wavebands from 0.6 to 7 µm, which is very close to the theoretically calculated transmittance (≈75%, which is calculated according to the Fresnel reflection theory with an average refractive index of 2.21 of the sample).^9^ The measured transmittance at 2.5 µm for the nanoceramics with the thickness of 1.5 mm is ≈67%, which is comparable to the precursor glass sample (≈70%) and ≈9.5 times larger than microceramics. The exceptional excellent optical transmission properties can be discussed based on the fundamental interaction mechanism between light and heterogeneous matter. According to Rayleigh–Debye theory, the turbidity which describes above interaction can be illustrated by the following equation^23^

(2) $$\tau \approx \frac{14}{15\pi }\varphi \left(1-\varphi \right)k^{8}R^{7}{\left(\frac{\Delta n}{\bar{n}}\right)}^{2}$$

where ϕ means the crystallization volume fraction, *k* represents the wave vector, and *k* = 2π/λ (λ is wavelength of the incident light), *R* means the average radius of the crystals, Δ*n* is the refractive index difference between the amorphous and crystalline phase, $\bar{n}$ is the average refractive index of the medium. The equation clearly indicates that the notable decrease in the crystal size and improvement of crystallinity can significantly depress the light scattering in the medium, thus resulting in the desired high transparency of the nanoceramics.

One of the prominent advantages of glass is that it belongs to the thermoplastic system. This feature provides an opportunity for fabrication of nanoceramic fiber in a continuous way, not feasible with conventional ceramic technology. We carried out tests by employing the modified fiber‐drawing technique. In details, the fiber elaboration was rationally separated in two steps: First, fiber‐drawing was processed under extremely low viscous state (≈100 Pa s), in order to prevent unexpected crystallization; second, the nanoceramics fiber were obtained via thermal activation, based on the established heat‐treatment procedure described above. Based on the careful optimization of drawing procedure, nanoceramics fiber with classic core‐cladding configuration can be successfully elaborated.

**Figure 5:**
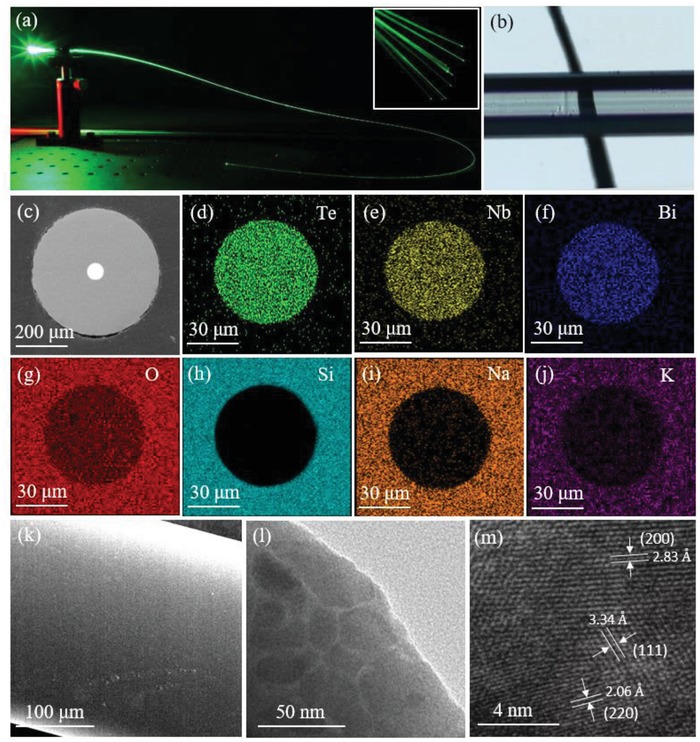
Elaboration of nanoceramics fiber. a) The photographs showing the nanoceramics pumped with 532 nm laser. b) The optical microscopy image of the bare nanoceramics fiber without cladding. c–j) The SEM image and EDS mapping of the nanoceramics fiber. k) The SEM image on the surface of a bare nanoceramics fiber without cladding. l) TEM and m) high‐resolution transmission electron microscopy (HRTEM) image of the nanoceramics fiber.

**Figure** **5**a presents a typical nanoceramics fiber coupled with the 532 nm laser beam, demonstrating the excellent light propagation property. The transmission microscopy image (Figure 5b) of a bare nanoceramics fiber without clad clearly shows the line on the substrate, further demonstrating the optical transparency of the nanoceramics fiber. The backscattered electron image (Figure 5c) and the corresponding energy‐dispersive spectra (Figure 5d–j) of the nanoceramics fiber with the standard core‐clad configuration clearly indicate that the constituent elements of nanoceramics are homogenously distributed in the core region of the fiber with the size of ≈65 µm. The superior optical quality and homogenous structure of nanoceramics fiber are believed to be mainly benefited from the extremely tiny size of the crystalline domains. As evidenced by the SEM image (Figure 5k) of a bare nanoceramics fiber without clad, the surface is highly smooth, that is an entirely different scenario form the case for the microceramics where rather rough surface is caused by the irregular distribution of microsized crystals (Figure S3, Supporting Information). Transmission electron microscopy (TEM) image indicates that nanoceramics fiber is embedded with densely stacked nanoparticles with the average size of ≈20 nm. Excitingly, the single particle is composed of extremely tiny crystalline domains with different *d*‐spacing of ≈2.83, 3.34, and 2.06 nm. This configuration is believed to be critical to avoid formation of unexpected imperfections such as cracks and inferior surface morphology, because these domains can rotate flexibly to accommodate surface‐tension induced transformation during fiberization, resulting in creation of optically superior fiber. Furthermore, this important finding provides additional evidence of the proposed nucleation dominated crystallization habit in nanoceramics.

**Figure 6:**
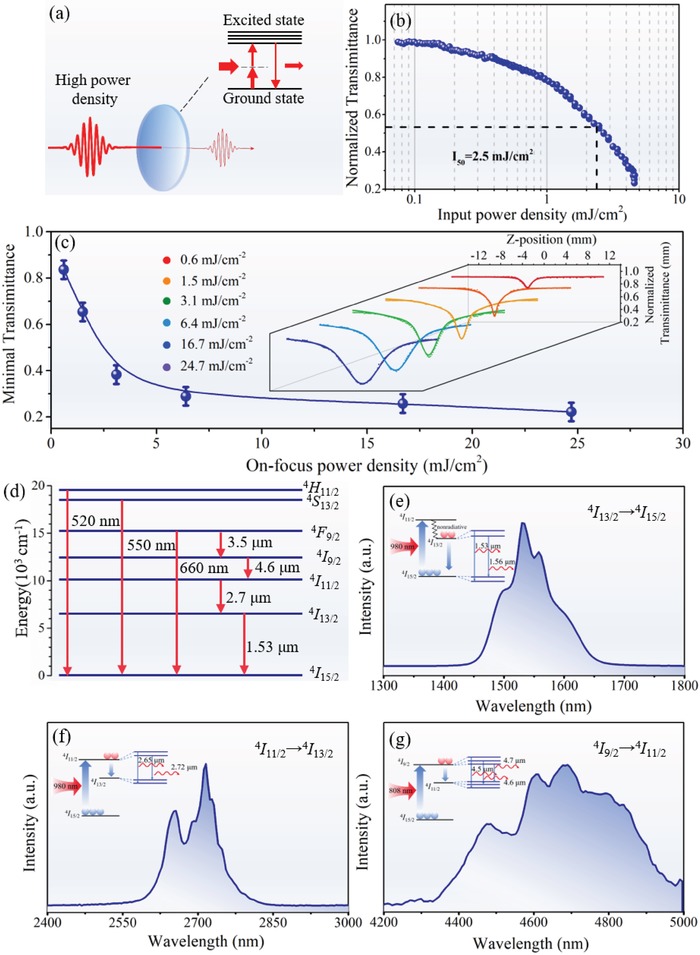
The photonic applications, including the dynamic radiation filtering and near‐/mid‐infrared radiative transition of the nanoceramics. a) The schematic nonlinear optical response. b) The input power density dependent normalized transmittance. c) The minimal transmittance as a function of the on‐focus power density. The inset shows the corresponding power dependence open aperture *Z*‐scan curves of the nanoceramics sheet with 0.5 mm thick. d) The energy level diagram of Er^3+^ ions. The near‐/mid‐infrared luminescence of Er^3+^‐doped nanoceramics under excitation with e,f) 980 nm and g) 808 nm.

The intriguing properties of the elaborated nanoceramics and fiber prompted us to explore their novel applications. Especially, the unique combination of main groups species (Bi^3+^ and Te^4+^) with high polarizability and transition metal ion (Nb^5+^) with empty d orbital is expected to gift the resultant material system with giant nonlinear optical response, allows for smart filtering high‐energy radiation. The basic mechanism involves high photon‐flux triggered multiphoton absorption. Under low luminous radiation, the nanoceramics are highly transparent to the arrived optical beam; Once the photon density reaches a threshold, two‐photon absorption will occur and its optical transmission will dramatically decrease in an automatic and ultrafast manner (**Figure** **6**a). To check above processes, the nonlinear optical properties of the nanocermaics were examined via employing the standard open‐aperture *Z*‐scan technique. As a typical measurement, a coherent femtosecond laser (center wavelength: 530 nm, pulse duration: 100 fs, 3 dB spectral width: 15 nm, and repetition rate: 1 kHz) was focused and passed through the sample. The transmittance is measured as a function of the input power density which is tuned by moving the samples close to or away from the focus (*Z* = 0) along light propagated direction. Figure 6b exhibits the laser power dependent transmittance on a thin sheet of the nanoceramics (≈0.5 mm). It can be found that the normalized transmittance significantly decreases with the increase of power, firmly indicating the occurrence of reverse saturable absorption. To vividly exhibit the input energy dependent transmittance, the minimal transmission (*T*_min_) as a function of on‐focus energy density was plots in Figure 6c. Notably, the sharp decreasing trend of *T*_min_ and the extremely low *T*_min_ (≈20%) at high radiation can be clearly observed. Two‐photon absorption (TPA) model can be used to fit above changes and the normalized transmittance *T*(*Z*) can be described by Equations (3) and (4), 24

(3) $$T\;\left(Z\right)=\sum_{m=0}^{\infty }\frac{{\left[-q_{0}\left(Z\right)\right]}^{m}}{{\left(m+1\right)}^{1.5}}$$

(4) $$q_{0}=\;\beta \frac{I_{0}\left[1-\exp\left(-\alpha_{0}L\right)\right]}{\left[1+{\left(Z/Z_{0}\right)}^{2}\right]\alpha_{0}}$$

where α_0_ and β are the linear absorption coefficient and the TPA coefficient, respectively, *L* is the thickness of the sample, Z_0_ = *kω* ^2^ is the diffraction length of beam, *k* = 2π/λ is the wave number, and ω is the beam waist radius. Based on these relations, the β parameter which directly reflects the TPA property can be calculated to be 3.065 cm GW^−1^, which is much larger than that of the other classic nonlinear candidates such as 0.565 cm GW^−1^ for TeO_2_–Bi_2_O_3_–WO_3_ system, 1.428 cm GW^−1^ for Bi_2_O_3_–B_2_O_3_–TiO_2_ system, and 1.483 cm GW^−1^ for TeO_2_–TiO_2_ system.^25^ We further calculated the limiting threshold which is regarded as the key indicator for evaluation of the radiation shielding performance. Encouragingly, the optical limiting threshold of nanoceramics was estimated to be 2.5 mJ cm^−2^, which is more than one order of magnitude smaller than the reported material system such as 117 mJ cm^−2^ for conjugated organic molecules, 74 mJ cm^−2^ for carbon nanodots, and 21.6 mJ cm^−2^ for WSe_2_.^26^ Above results firmly demonstrate the promising application of nanoceramics for dynamic radiation shielding.

Another key advantage of the constructed multicomponent nanoceramics is the great capability for host various active dopants. As a proof‐of‐concept test, erbium (Er^3+^) was selected as the active dopant, taking into account its rich potential electronic transitions (Figure 6d) and essential roles in photonics.^27^ Encouragingly, Er^3+^‐doped nanoceramics exhibits broadband ^4^ *I*_13/2_→^4^ *I*_15/2_ radiative transitions at 1450–1650 nm with the full width at half‐maximum (FWHM) of 83 nm (Figure 6d), which is more than two times larger than that of the conventional glass fiber amplifier (≈30 nm). The extension of bandwidth indicates its potential application as gain media for optical amplifier in broadband telecommunication system.^28^ Furthermore, it also shows intense mid‐infrared luminescence at 2550–2850 and 4300–4950 nm (Figure 6f,g), which are originated from ^4^ *I*_11/2_→^4^ *I*_15/2_ and ^4^ *I*_9/2_→^4^ *I*_11/2_ electronic transitions.^29^, ^30^ It is necessary to note that the past researches about the mid‐infrared luminescence at 4300–4950 nm are strictly limited to the chalcogenide and halogenide host.^31^, ^32^, ^33^ To the best of our knowledge, it is the first time to report the radiative transition beyond 4300 nm in Er^3+^‐doped oxide nanoceramics system. In comparison to the chalcogenide and halogenide host, the oxide system exhibits several prominent advantages such as high chemical stability and easy fabrication. We further confirmed that the present nanoceramics can host other types of dopants such as Co^2+^ and Ni^2+^, and produce various unique electronic transitions (Figure S4, Supporting Information). The interesting optical phenomena are believed to be benefited from the cooperative factors including special structural units, moderate phonon energy (≈750 cm^−1^), and superior optical transmittance in the infrared waveband (from 0.6 to 7 µm) offered by heavy‐metal species which are favorable for supporting radiative transition in nanoceramics. Above results indicate that the constructed telluritebased nanoceramics in this work should be the promising active candidate as near‐/mid‐infrared amplifiers and lasers.

## 3 Conclusion

In summary, we demonstrate our pressureless phase‐transition approach toward transparent nanoceramics. The novelty of the proposed method is embodied in the creation of a sharp contrast between nucleation and growth tendency, which move beyond a conventional crystallization regime, and thereby enable a vastly expanded parameter space for tuning the microstructures of crystallized glass. This work represents the first proof‐of‐principle dense nanoceramics and smooth fibers with optical properties including the dynamic optical filtering ability and efficient radiative transition in the mid‐infrared wavebands. The knowledge gained from our theoretical and experimental studies will allow for creation of next‐generation fibers that may revolutionize the fields of lasers, high‐energy ray/particle detection, and green lighting.

## 4 Experimental Section

*Material Synthesis*: Glass with a composition of 75TeO_2_–*x*Bi_2_O_3_–(25−*x*)Nb_2_O_5_ (11.5 ≤ *x* ≤ 18.5) (in mol%) was prepared by the melt‐quenching technique. The TeO_2_ (aladdin, 99.999%), Bi_2_O_3_ (aladdin, 99.99%), and Nb_2_O_5_ (aladdin, 99.9%) were used as raw materials. After mixing thoroughly, the materials were put in the alumina crucibles and then kept at 850 °C for 15 min. The melt was poured into a mold and then pressed with a brass plate to form the parent glass. The samples were annealed at 300 °C for 4 h to relieve the internal stress. The nanocrystallization process was conducted at a fixed heat‐treatment temperature. The highly crystallized and transparent nanoceramics were obtained after heat‐treating at 440 °C for 1.5 h, based on the composition of 75TeO_2_–16.5Bi_2_O_3_–8.5Nb_2_O_5_ (in mol%). The nanoceramics fiber was fabricated via a rod‐in‐tube method. The matrix glass rod was inserted into the tube made of K9 glass. The fiber was drawn at 950 °C with the drawing speed of 2 m min^−1^. The nanocrystallization of fiber was achieved by the same hear‐treatment procedure similar to bulk nanoceramics.

*Characterization Methods*: Thermal studies were performed on a Netzsch STA449C simultaneous thermal analyzer. Crystalline phase was characterized by XRD analysis on an X‐ray diffractometer, using Cu/Ka as radiation source. The crystallinity can be calculated based on DSC analysis or XRD patterns.^34^, ^35^ In this work, the crystallinity was evaluated by Rietveld analysis based on the XRD patterns. The microstructures of the sample were studied using a ZEISS Merlin field‐emission scanning electron microscope with an energy‐dispersive spectrometer and a JEM‐2100F transmission electron microscope. The optical transmission spectra at the wavebands of 200–2500 and 2500–8000 nm were measured on a Lambda‐950 ultraviolet (UV)/visible (vis)/near‐infrared (NIR) spectrophotometer and Vector 33‐MIR fourier transform infrared spectrometer, respectively. The refractive index was characterized on a MODE12010/M prism coupler. The Raman measurements were carried out on an INVIA laser micro‐Raman spectrometer. The mechanical properties were measured using a HVS‐10 Vickers hardness tester and an Anton Paar TTX‐NHT3 nanoindentator equipped with a diamond Berkovich indenter. A constant load of 5000 µN with a 10 s dwell time at maximum depth was applied to the polished surface of the samples. The nonlinear optical properties were investigated by Z‐scan measurements on a Coherent femtosecond laser focused by the lens with the focal length of ≈500 mm. The incident light was centered at 800 nm with a pulse duration of 100 fs and repetition rate of 1 kHz. The infrared fluorescence spectrum were characterized on a OMNI5015i infrared fluorescence spectrometer equipped with the InSb detector and SR830 phase‐locking amplifier, using 808 and 980 nm laser as the pumping sources.

*Theoretical Analysis*: The probability distribution of single phase formation under different chemical potential was calculated via combining the grand canonical partition function and the expectation value of particles under the microscopic state, based on the grand canonical ensemble of thermodynamics and statistical physics.

## Conflict of Interest

The authors declare no conflict of interest.

## Supporting information

Supplementary
