# Supplementary material for: Pressureless Crystallization of Glass for Transparent Nanoceramics
Source: Adv Sci (Weinh). 2019 Jun 28;6(17):1901096. doi: 10.1002/advs.201901096 (PMC6724475; doi:10.1002/advs.201901096)
Supplement: Supplementary file 1 — Supplementary [file ADVS-6-1901096-s001.pdf]

Copyright WILEY-VCH Verlag GmbH & Co. KGaA, 69469 Weinheim, Germany, 2019.

Supporting Information

for *Adv. Sci.,* DOI: 10.1002/advs.201901096

Pressureless Crystallization of Glass for Transparent Nanoceramics

*Shaofei Wen, Yunpeng Wang, Bijiao Lan, Weida Zhang, Zhuo Shi, Shichao Lv, Yujun Zhao, Jianrong Qiu, and Shifeng Zhou**

Supporting Information

Pressureless crystallization of glass for transparent nanoceramics

*Shaofei Wen, Yunpeng Wang, Bijiao Lan, Weida Zhang, Zhuo Shi, Shichao Lv, Yujun Zhao, Jianrong Qiu and Shifeng Zhou**

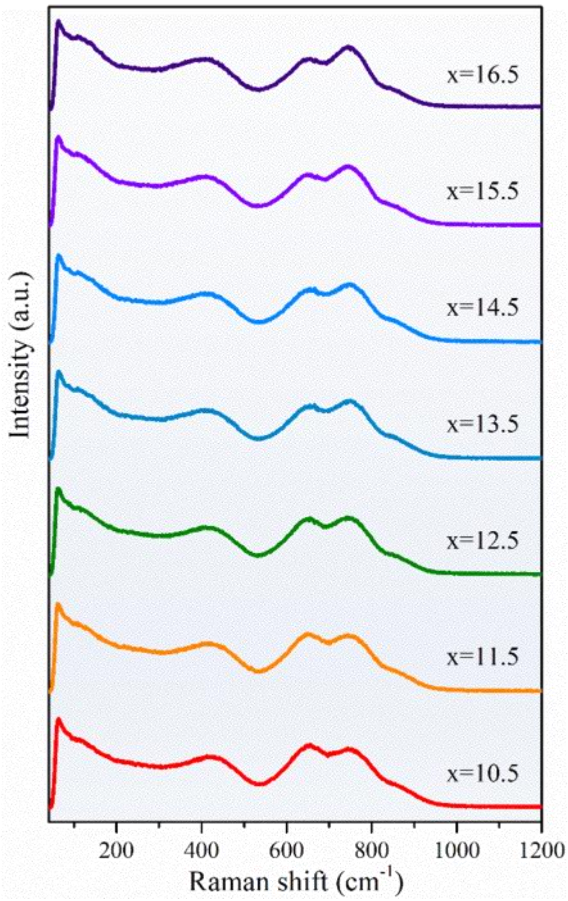

Figure S1 The Raman spectra of the parent glass with the composition of $75TeO_2$-xBi_2O_3-(25-x)Nb_2O_5 parent glass(in mol%). The presence of Raman peak at the low wavenumber (<100 cm^{-1}) and the shoulder at around 120 cm^{-1} indicates the Bi^{3+} ions occurs in the form of [BiO_6] and [BiO_3] groups. With the increase of Bi_2O_3 contents, these two bands separated gradually and the latter become obvious, which can be attributed to the increasing of [BiO_6] and [BiO_3] groups. The band at around 430 cm^{-1} associated with the Te-O-Te linkages shifts to lower frequency waveband, illustrating that the Te-O-Te linkages are replaced gradually by the weaker Te-O-Bi or Bi-O-Bi linkages. Besides, with the replacing of TeO_2 by Bi_2O_3, the change of the peak at ~656 cm^{-1}(TeO_4) and ~753 cm^{-1} (TeO_{3+1} or Te=O) can be assigned to the transition of $TeO_4 \rightarrow TeO_{3+1} \rightarrow$ Te=O.

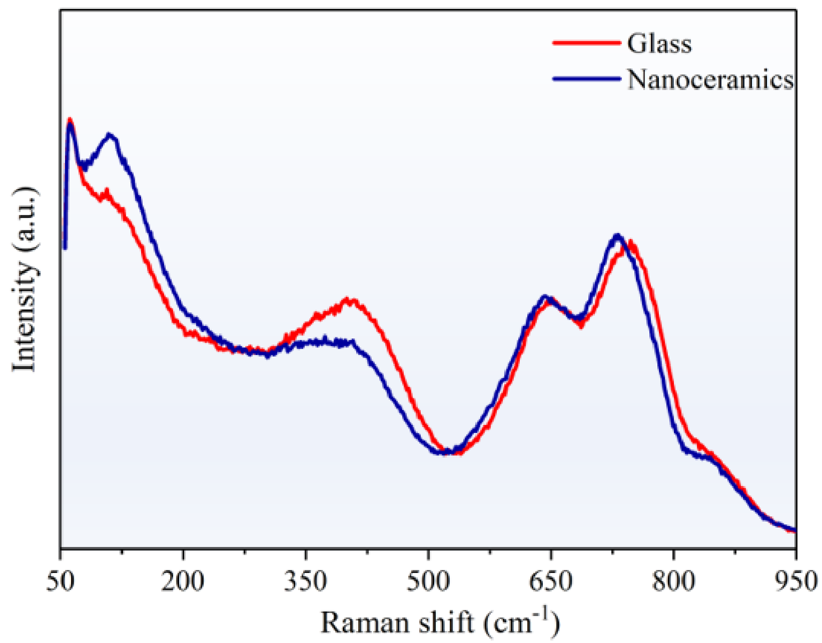

Figure S2. Raman spectra of as-made glass and nanoceramics. The stretching vibration of Bi-O linkages at around 110 cm^{-1} can be obviously observed in the nanoceramics. The peak at around ~400 cm^{-1} which is associated with the Te-O-Bi linkages becomes weaker and shifts to the lower frequency waveband in the nanoceramics, indicating that more Bi displaces the site of Te in Te-O-Bi linkages. Besides, the stretching vibration of Nb-O (at around 842 cm^{-1}) becomes prominent in the nanoceramics.

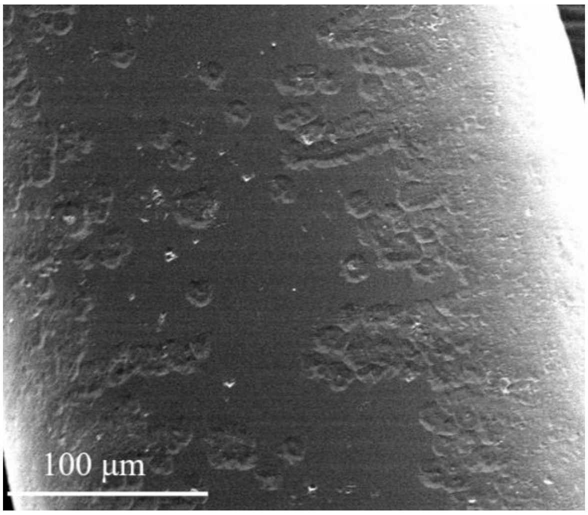

Figure S3 SEM image of the bare fiber of Bi12.5 after crystallization.

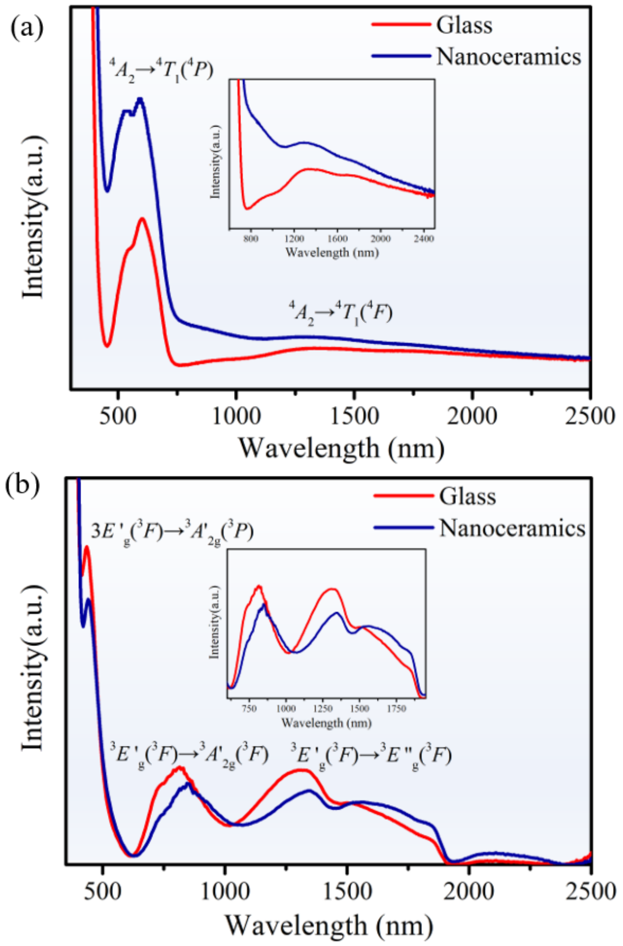

Figure S4 Optical performance for transition metal ions dopants. (a) The absorption spectra of the glass and nanoceramics doped with 1 mol% Co^{2+}. The inset shows the enlarged region of the near-infrared waveband. The absorption at 500-750 nm is abtributed to the $^4A_2\rightarrow^4T_1(^4P)$ transition. The broad near-infrared absorption is related to the $^4A_2\rightarrow^4T_1(^4F)$ transition. A shoulder located at around 540 nm and the extension of the 500-700 nm band can be clearly observed in the nanoceramics. The difference in the near-infrared absorption between the parent glass and nanoceramics indicates the doping of Co in nanoceramics. (b) The absorption spectra of the glass and nanoceramics doped with 1 mol% Ni^{2+}. The inset shows the enlarged region of the near-infrared waveband. The absorption band centered at ~435, ~820 and ~1310 nm can be assigned to the $^3E'_g(^3\mathrm{F})\rightarrow^3A'_{2g}(^3P)$, $^3E'_g(^3F)\rightarrow^3A'_{2g}(^3F)$ and $^3E'_g(^3F)\rightarrow^3E''_g(^3F)$ transition, respectively. A notable red-shift for the absorption band at ~820 and ~1310 nm can be observed in the nanoceramics, indicating the incorporation of Ni^{2+} ions in nanoceramics.
